# *Mycobacterium vaccae* Nebulization Can Protect against Asthma in Balb/c Mice by Regulating Th9 Expression

**DOI:** 10.1371/journal.pone.0161164

**Published:** 2016-08-12

**Authors:** Chaoqian Li, Xiaohong Jiang, Mingjie Luo, Guangyi Feng, Qixiang Sun, Yiping Chen

**Affiliations:** 1 Department of Respiratory Medicine, Guangxi Medical College, Nanning, Guangxi, China; 2 Department of Geriatric Respiratory Medicine, The First Affiliated Hospital of Guangxi Medical University, Nanning, Guangxi, China; 3 Department of Respiratory Medicine, The First Affiliated Hospital of Guangxi Medical University, Nanning, Guangxi, China; 4 The Graduate School of Guangxi Medical University, Nanning, Guangxi, China; 5 Department of Geriatric Disease, The National Affiliated Hospital of Guangxi Medical University, Nanning, Guangxi, China; Kyoto Daigaku, JAPAN

## Abstract

Asthma is a heterogeneous disease characterized by chronic airway inflammation. CD4(+) T-helper 9 (Th9) cells are closely linked to asthma, helping to regulate inflammation and immunity. Epidemiological studies showed that mycobacteria infections are negatively associated with asthma. Our previous research showed that inactivated *Mycobacterium phlei* nebulization alleviated the airway hyperresponsiveness and inflammation of asthma. However, the relationship between Th9 cells and mycobacteria remains unknown. Here, we evaluated the relationship between *Mycobacterium vaccae* nebulization and Th9 cells in asthmatic mice. Eighteen Balb/c mice were randomized into 3 groups of 6 mice each (normal control group, asthma control group, and nebulization asthma group [Neb. group]). The Neb. group was nebulized with *M*. *vaccae* one month before establishment of the asthmatic model with ovalbumin (OVA) sensitization, and the normal and asthma control groups were nebulized with phosphate-buffered saline. The hyperresponsiveness of the mouse airways was assessed using a non-invasive lung function machine. Lung airway inflammation was evaluated by hematoxylin and eosin and periodic acid-Schiff staining. Cytokine interlukin-9 (IL-9) concentration and OVA-specific IgE in the bronchoalveolar lavage fluid were measured by enzyme-linked immunosorbent assays. The percentages of γδTCR^+^ CD3^+^, IL-9^+^CD3^+^, IL-10^+^CD3^+^ lymphocytes, and IL9^+^γδT and IL-10^+^γδT cells were detected by flow cytometry. The airway inflammation and concentration of IL-9 and OVA-specific IgE were significantly reduced in the Neb. group compared to the asthma control group. The Neb. group had lower airway hyperresponsiveness, percentages of γδTCR^+^CD3^+^ and IL-9^+^CD3^+^ lymphocytes, and IL9^+^γδT cells, and higher percentages of IL-10^+^CD3^+^ lymphocytes and IL-10^+^γδT cells compared to the asthma control group. Thus, mouse bronchial asthma could be prevented by *M*. *vaccae* nebulization. The mechanism could involve *M*. *vaccae*-mediated effects on induction of IL-9 secretion and suppression of IL-10 secretion from γδT cells. γδT cells showed prominent IL-10 expression, indicating that they possibly belong to the Th9 family.

## Introduction

Bronchial asthma is a common chronic respiratory disease affecting all age groups. Its prevalence is increasing in many countries, especially among children. Although some countries have witnessed a decline in hospitalization and deaths from asthma, it still imposes an unacceptable burden on healthcare systems and on society through loss of productivity in the workplace, especially pediatric asthma. Asthma is characterized by variable symptoms, including wheezing, shortness of breath, chest tightness and/or cough, and variable expiratory airflow limitation [1]. Although these symptoms can be controlled, long-term relapse has been shown to contribute to airway remodeling and the development of chronic obstructive pulmonary disease.

Asthma is a heterogeneous disease, usually characterized by chronic airway inflammation [1], and some T lymphocytes such as helper T lymphocytes (Th) and regulatory T cells (Tregs) affect the inflammation duration. Specifically, there is an increase in Th2 and Th17 cells, and a decrease in Th1 and Treg cells in asthma. Th9 cells, a new type of CD4^+^T lymphocytes, mainly secrete interleukin (IL)-9 and IL-10, which are closely linked with asthma [2, 3]. The quantity of Th9 cells correlates directly with the severity of airway hyperresponsiveness and airway inflammation, which can be alleviated by anti-IL-9 treatment [4]. The Th9 cell is a novel CD4^+^ effector T cell, characterized by IL-9 and -10 dominant secretion [5]. Cytokine IL-9, formerly named P40, is a T cell growth factor, and Th9, Treg, Th1, and Th17 cells can produce cytokine IL-9, which was previously classified as a Th2 type cytokine. IL-9 is a multi-effector cytokine acting on various inflammatory and tissue cells, producing various biological effects, and is closely associated with asthma. Cytokine IL-10, produced predominantly by Th2 and Treg cells, is an important suppressive cytokine, which can inhibit T cell proliferation and differentiation, as well as inflammation [6]. Research has shown that the concentration of IL-10 in asthmatic sufferers was obviously higher than that in healthy subjects [7]. Exogenous IL-10 intratracheal instillation can alleviate airway inflammation [8]. Other studies have shown that Th9 cells can promote allergic reactions, activate mast cells, and aggregate pathological manifestation; adoption of Th9 cells in mice contributed to allergic airway inflammation, while anti-IL-9 treatment could alleviate airway inflammation as well as decrease airway hyperresponsiveness [9, 10]. A major characteristic of allergic asthma is the increase of allergen-specific immunoglobulin E (IgE), which can promote airway hyperresponsiveness. γδT cells, important immunocytes that are extensively present in the skin and mucosal tissue, occupying 10–50% of the epidermal and mucosal epithelia and 10–30% of pulmonary tissues, can regulate airway hyperresponsiveness [11], restrain airway inflammation [12], and play the role of antigen-presenting cells [13]. Another study showed that γδT cells participate in the development of asthma through the release of Th1/Th2/Th17 cytokines [14].

Asthma can be treated and controlled in most sufferers effectively; nevertheless, the etiology and pathogenesis of asthma are still unknown, and there are no efficient prevention measures. Epidemiological studies showed that *Mycobacterium* infections inhibit the development of asthma [15, 16]. Our previous studies showed that inactivated *Mycobacterium phlei* nebulization could alleviate the airway hyperresponsiveness of asthmatic sufferers, and the airway inflammation in asthmatic mice [17, 18]. The attenuated live vaccine Bacillus Calmette-Guerin (BCG), belonging to *Mycobacterium bovis*, was found to be unstable when used in clinical studies for asthma prevention [19], but animal experiments demonstrated that BCG could protect against an asthmatic model in rats [20] by restraining the airway remodeling of asthma [21]. *Mycobacterium vaccae*, an avirulent mycobacterium, is commonly used as adjuvant therapy for tuberculosis [22–24]. Nebulization therapy is important in respiratory disease treatment and has the following advantages: the medicine can reach the pulmonary mucous membrane surface directly to undertake mucosa immunity immediately, it can be simply and easily performed, and there are few side effects.

To date, there have been no reports on the prevention of asthma by *M*. *vaccae*, or on the relationship between *M*. *vaccae* and Th9 cells. Based on the foundation of our previous research, we determined the effect of nebulized *M*. *vaccae* in asthmatic mice to explore the effect of *M*. *vaccae* on asthmatic prevention and Th9 cells. Therefore, the aim of the current study was to investigate the effect of *M*. *vaccae* on the prevention of asthma.

## Materials and Methods

### Ethics statement

The study was performed in accordance with the Guide for the Care and Use of Laboratory Animals of the National Institutes of Health, and was approved by the Guangxi Medical University Animal Care and Use Committee (Protocol number: 20131002). All surgeries were performed under nembutal anesthesia and all efforts were made to minimize suffering.

### Animals

Eighteen 8–10-week-old pathogen-free female Balb/c mice (20–25 g) were provided by the Medical Animal Center of Guangdong Province (Guangdong, China). They were maintained in an air-conditioned room (temperature: 23 ± 3°C, humidity: 55.5 ± 1%). Animals were fed ovalbumin (OVA)-containing pellet feed and water *ad libitum*. Animals were also monitored daily for signs of respiratory distress, and were euthanized once this was detected (although no such action was necessary).

### Experimental protocol

**Fig 1:**
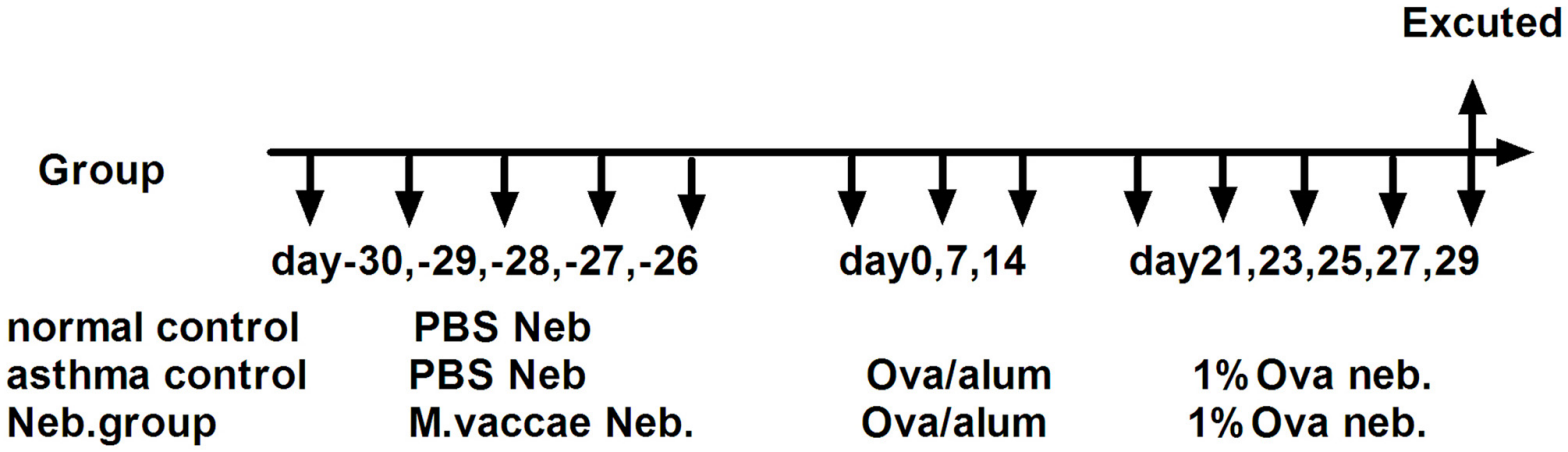
Experimental protocol. *M*. *vaccae*: *Mycobacterium vaccae*; Neb.: nebulization.

Mice were randomized into three groups: normal, asthma control, and nebulization prevention (Neb. group). Before the asthmatic model was established, the Neb. group was nebulized with 22.5 μg *M*. *vaccae* (Anhui Longkema Biological Pharmaceutical Co., Anhui, China) mixed with 10 mL phosphate buffered saline (PBS) once daily for 5 consecutive days. The normal and asthma control groups were nebulized with PBS. The asthmatic model was improved according to the method of Gorman et al. [25]. In brief, each mouse was injected intraperitoneally with an OVA (Grade V, Sigma, St. Louis, MO, USA) mixture (20 μg OVA, 1 mg aluminum hydroxide gel [Pierce, Waltham, MA, USA), 200 μL PBS) once weekly for 3 consecutive weeks. From week 4, they were nebulized with 1% OVA/PBS liquid (1g OVA in 100 mL PBS) 30 min daily for 5 consecutive days (Fig 1).

### Airway responsiveness measurement

Airway responsiveness was measured using a non-invasive lung function machine (FinePointe^™^ NAM system TBL4500, Buxco, Wilmington, NC, USA) after the last stimulation with OVA. After calibration and 5 min of adaptation, the mice were nebulized with 20 μL PBS and methacholine (Mch; Sigma) at concentrations of 6.25, 12.5, and 25 mg/mL, respectively, for 30 s each. Data were recorded for 3 min and the mice were allowed to recover for 4 min. The results were analyzed automatically after the experiment ended. The airway responsiveness is presented as the expiratory flow at 50% tidal volume (EF50).

### Specimen collection

The mice were anesthetized using an intraperitoneal injection of 1% nembutal (50 mg/kg body weight), dehematized through their eyeballs, and fixed on their backs. The lungs were lavaged using 500 μL iced PBS three times, and the bronchoalveolar lavage fluid (BALF) was collected in EP tubes on ice. The recovery of BALF was >80%. The BALF was centrifuged to identify cytokines in the supernatant. The right upper lobes were fixed in formalin for histology and the left lobes were cut into pieces and digested with 0.1% collagenase IV (Sigma) for 45 min to produce a single cell suspension. Lymphocytes in the single cell suspension were separated using mouse tissue lymphocytes separation liquid (Haoyang, Tianjing, China) according to the operating instructions for cytometry analysis.

### Histology

Formalin-fixed (10%), paraffin-embedded lung sections of 3 mm were stained with hematoxylin and eosin (HE) and Periodic acid-Schiff (PAS) for morphological evaluation of the lung tissue.

### Enzyme-linked immunosorbent assay (ELISA) for IL-9 and OVA-specific IgE in BALF

The supernatant collected from the BALF was used to measure IL-9 and OVA-specific IgE levels using an anti-mouse IL-9 ELISA kit (RayBiotech, Norcross, GA, USA), anti-mouse OVA-specific IgE ELISA kit (BioLegend, San Diego, CA, USA), and anti-mouse IL-10 ELISA kit (eBioscience, San Diego, CA, USA), following the manufacturer instructions. The 450 nm optical density (OD) value was detected by ELISA after the termination reaction, and the cytokine IL-9 and OVA-specific IgE concentrations were defined by comparison between the OD value and the standard curve.

### Flow cytometry

Collected lymphocytes were added to PBS containing fetal bovine serum to make a 500 μL lymphocyte suspension, and 2 μL PMA(Phorbol-12-myristate-13-acetate)/ionomycin/BFA(Brefeldin A)/monensin mixture was added. The suspension was incubated for 4 h at 37°C in a 5% CO_2_ incubator, and the 500 μL lymphocyte suspension was divided into positive and control tubes (100 μL each). Anti-mouse-CD3-PE-CY5 (eBioscience) was added to all tubes; anti-mouse-gamma delta TCR-fluorescein isothiocyanate (FITC) (eBioscience) was added to positive tubes and Armenian Hamster IgG-FITC (eBioscience) was added to negative tubes. All tubes were incubated in the dark at room temperature for 30 min, and then 100 μL Reagent A (fixation medium, eBioscience) was added, and the tubes were incubated for 15 min at room temperature, washed, centrifuged for 5 min, and the supernatant was aspirated and vortexed to fully resuspend the cell pellet. Then, 100 μL Reagent B (permeabilization medium, eBioscience) was added to all tubes; 5 μL anti-mouse IL-9-PE (eBioscience) was added to positive tubes, IL-10-PE intracellular antibody (eBioscience) was added to the negative tubes, and the corresponding isotype was added to the control (eBioscience). All tubes were vortexed for 1 s and incubated for 20 min at room temperature in the dark. They were then washed with PBS containing 5% fetal bovine serum, centrifuged, and the supernatant was aspirated and detected after addition of 100 μL PBS. Cell populations were analyzed by flow cytometry with the EPICS XL system (Beckman-Coulter, Fullerton, CA, USA), and data analysis was performed with FlowJo software (ThreeStar, San Carlos, CA, USA).

### Statistical analysis

SPSS19.0 was used to calculate the standard deviations between experimental samples when each experimental group contained an equal number of data sets. Where different numbers of data sets existed in each experimental group, the standard error of the mean was used. When data were normally distributed and when two independent variables were analyzed, one-way ANOVA with Bonferroni post-hoc analysis was performed. In all other instances, statistical differences between groups were calculated using the Student’s t-test, with *P* < 0.05 considered significant. Graph Pad Prism 5.0 was used to produce graphs.

## Results

### Asthma symptom manifestation

No animals died before the mice were killed. During nebulization, mice in the asthma control group showed the following symptoms: agitation, scratching their ears and cheeks, forelimb crispation, shortness of breath, cyanosis of the oral lips and forelimbs, and urinary and fecal incontinence. Mice in the *M*. *vaccae* group showed none of the above manifestations.

### Mouse hyperresponsiveness after *M*. *vaccae* nebulization

**Fig 2:**
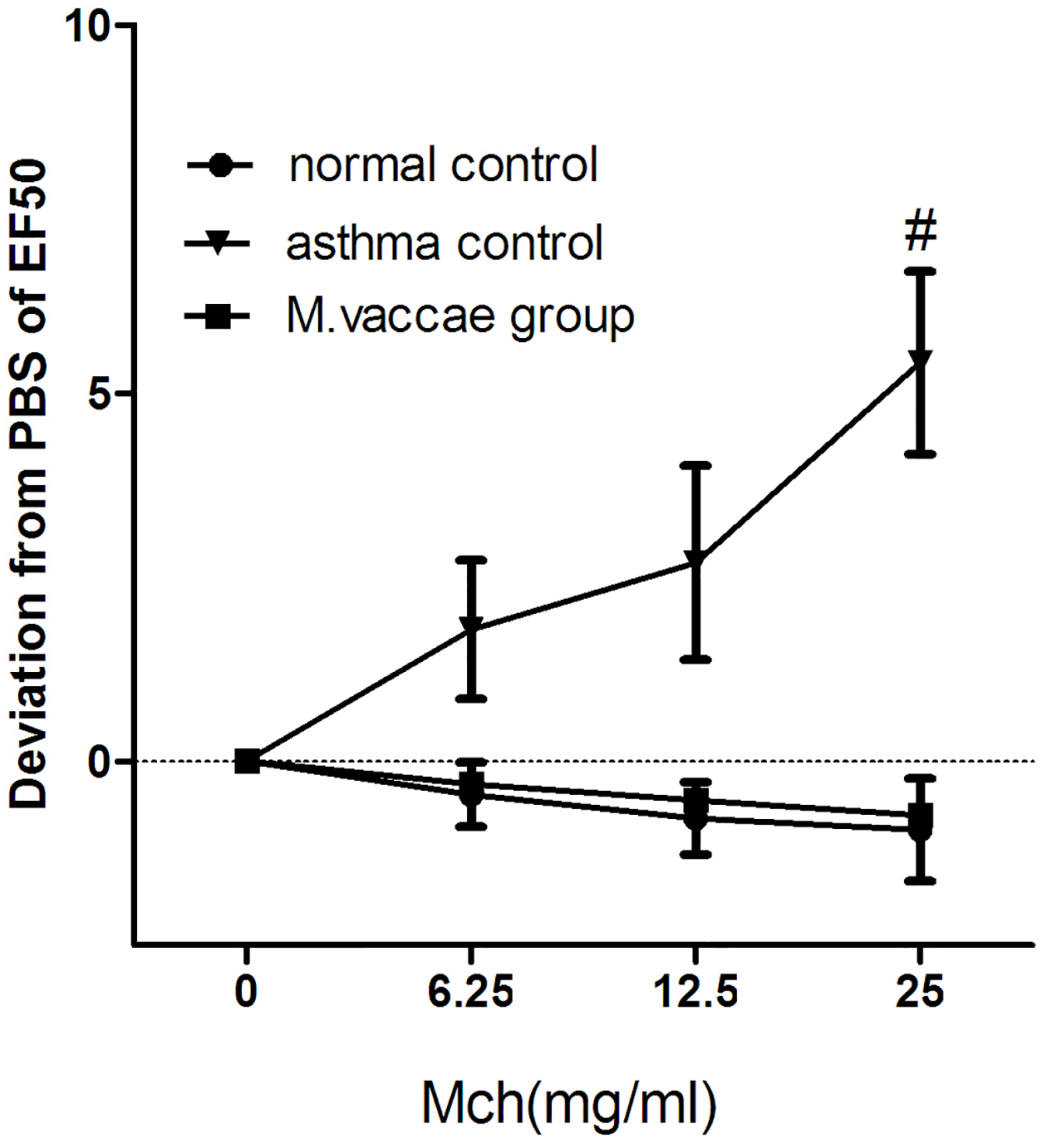
Airway hyperresponsiveness. ^#^*P* < 0.05, *M*. *vaccae* group vs. normal control group.

The OVA challenge significantly increased the responsiveness to Mch (Fig 2, *P* < 0.05), and *M*. *vaccae* nebulization decreased airway hyperresponsiveness sharply (Fig 2, *P* < 0.05).

### Histology

**Fig 3:**
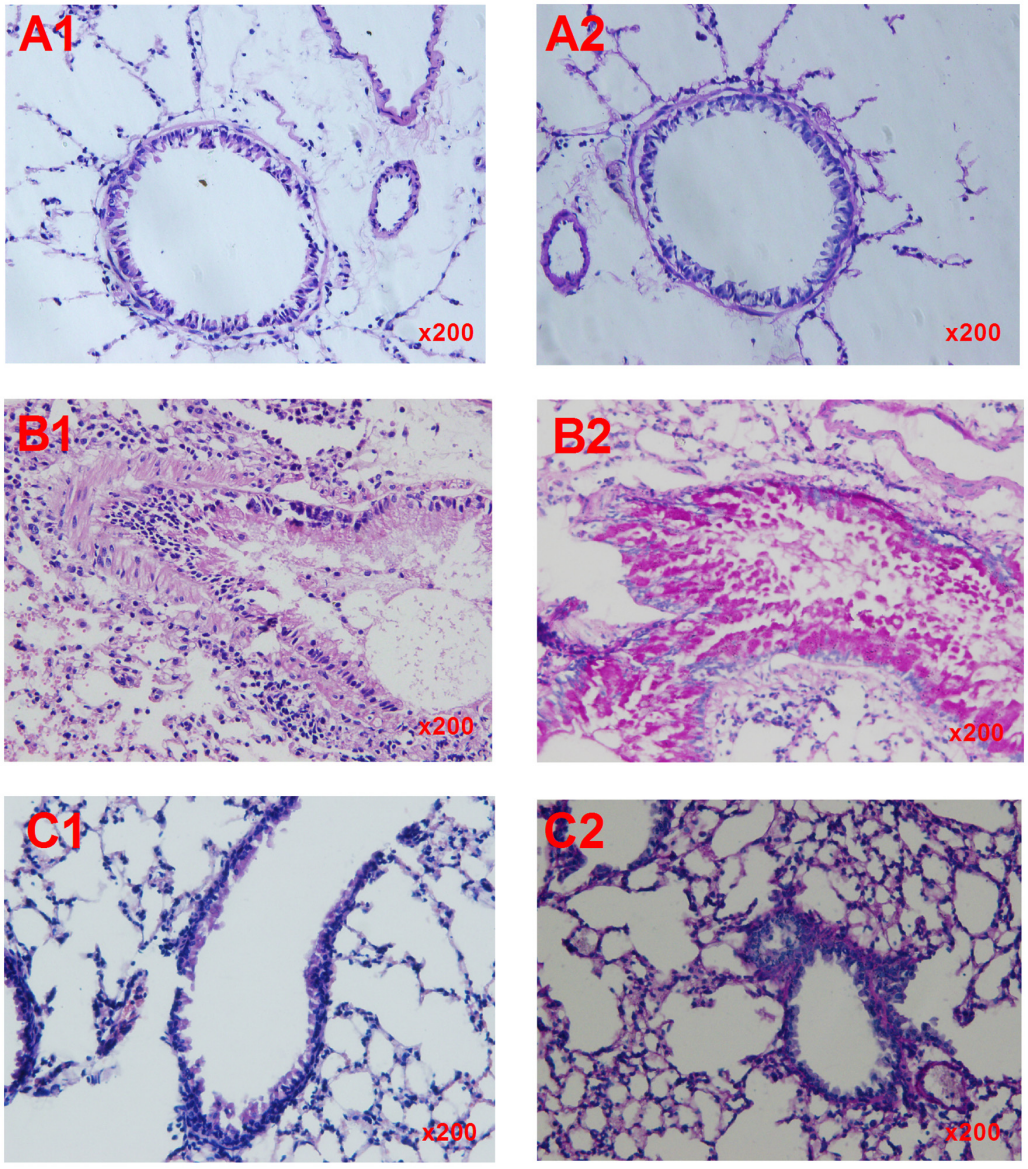
Lung histopathology. Lung histopathology was examined by hematoxylin and eosin (HE) staining and periodic acid-Schiff (PAS) staining in (A1 and A2) mice in the normal control group, (B1 and B2) mice in the asthma control group, and (C1 and C2) mice in the *M*. *vaccae* prevention group. The normal control group showed complete airway epithelium mucosa, ordered cilia, thin basilar membranes and smooth muscle, a smooth and glossy lung small vascular endothelium, no inflammatory cell infiltration, few goblet cells (A1), and no mucus secretion (A2). The asthma control group showed cellular swelling of the airway epithelia, increased plicae mucosae, disordered cilia, broken bronchial mucous membranes, infiltration of inflammatory cells, a thickened alveolar wall and basilar membrane, hyperplasia of the smooth muscle (B1), proliferation and hypertrophy of the airway epithelia and goblet cells, generous mucus and mucus plugs, and desquamation of the epithelia (B2). The *M*. *vaccae* group showed no obstruction of the airway lumen and ordered epithelia (C1), and no goblet cells or mucus secretion (C2).

The lungs in the normal control group mice showed complete airway epithelium mucosa, the cilia were ordered, the basilar membranes and smooth muscle were thin, the lung small vascular endothelium was smooth and glossy, there was no inflammatory cell infiltration around the vessels and airways, there were few goblet cells, and no mucus secretion (Fig 3A1 and 3A2). By contrast, in the asthma control group, there was cellular swelling of the airway epithelia, the plicae mucosae were increased, cilia were disordered, the bronchial mucous membranes were broken, many inflammatory cells infiltrated the bronchioles, the vessels had an alveolar space and interalveolar septum, the alveolar wall was thickened showing hyperemia and dropsy, the basilar membrane was thickened, the smooth muscle showed hyperplasia, and PAS staining showed airway epithelia and goblet cell proliferation and hypertrophy, generous mucus and mucus plugs, and desquamation of the epithelia (Fig 3B1 and 3B2). In the *M*. *vaccae* prevention group, the airway lumen was unobstructed and the epithelia lined up in order; PAS staining showed no goblet cells or mucus secretion (Fig 3C1 and 3C2).

### IL-9 and OVA-specific IgE levels in the BALF of *M*. *vaccae* prevention group mice

**Fig 4:**
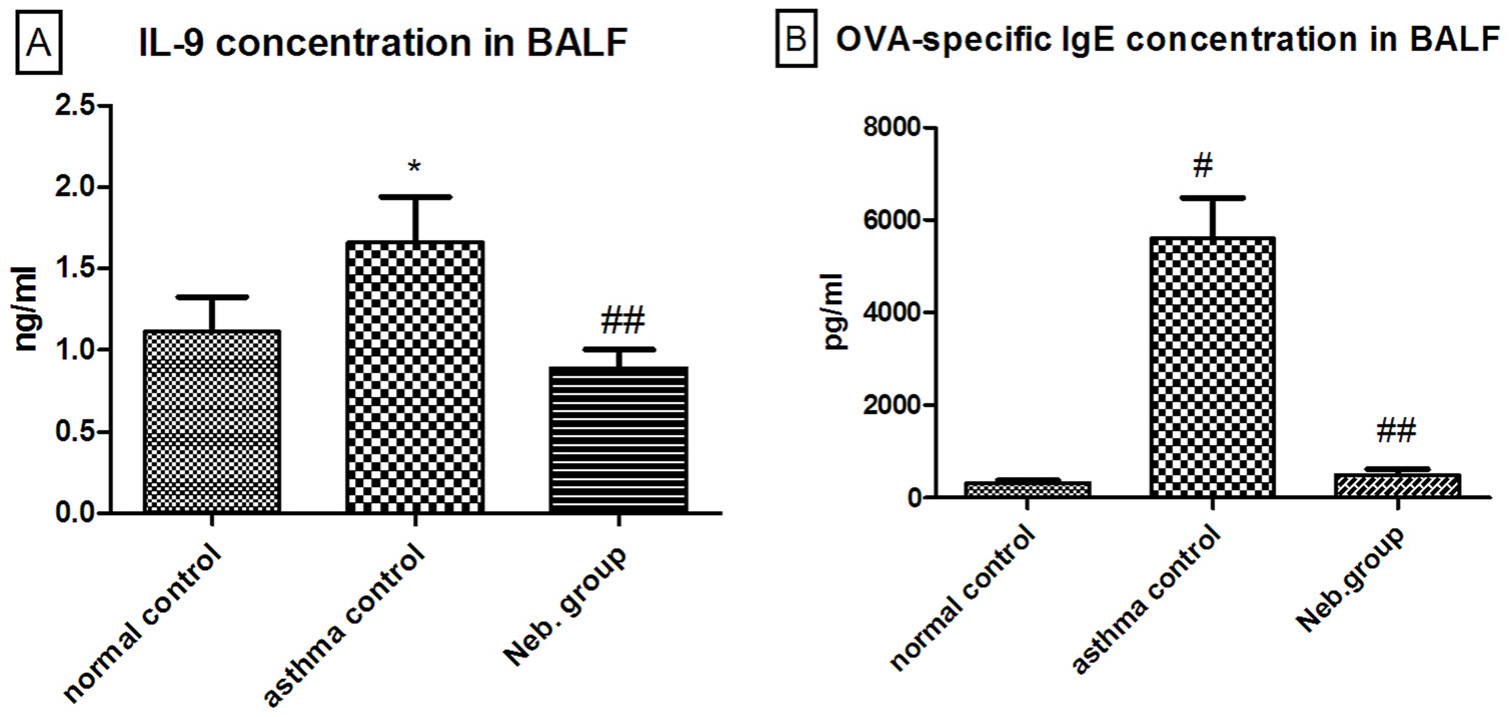
Concentration of cytokine IL-9 (A) and OVA-specific IgE (B) in the BALF. *P < 0.01, compared with the normal control; #*P* < 0.001, compared with the normal control; ##*P* < 0.001, compared with the asthma control.

OVA challenge significantly increased the levels of IL-9 and OVA-specific IgE in mice, but *M*. *vaccae* nebulization decreased the levels of both IL-9 and OVA-specific IgE (Fig 4A and 4B).

### The effect of *M*. *vaccae* nebulization on γδTCR^+^CD3^+^, IL-9^+^CD3^+^, IL-9^+^γδT lymphocytes

**Fig 5:**
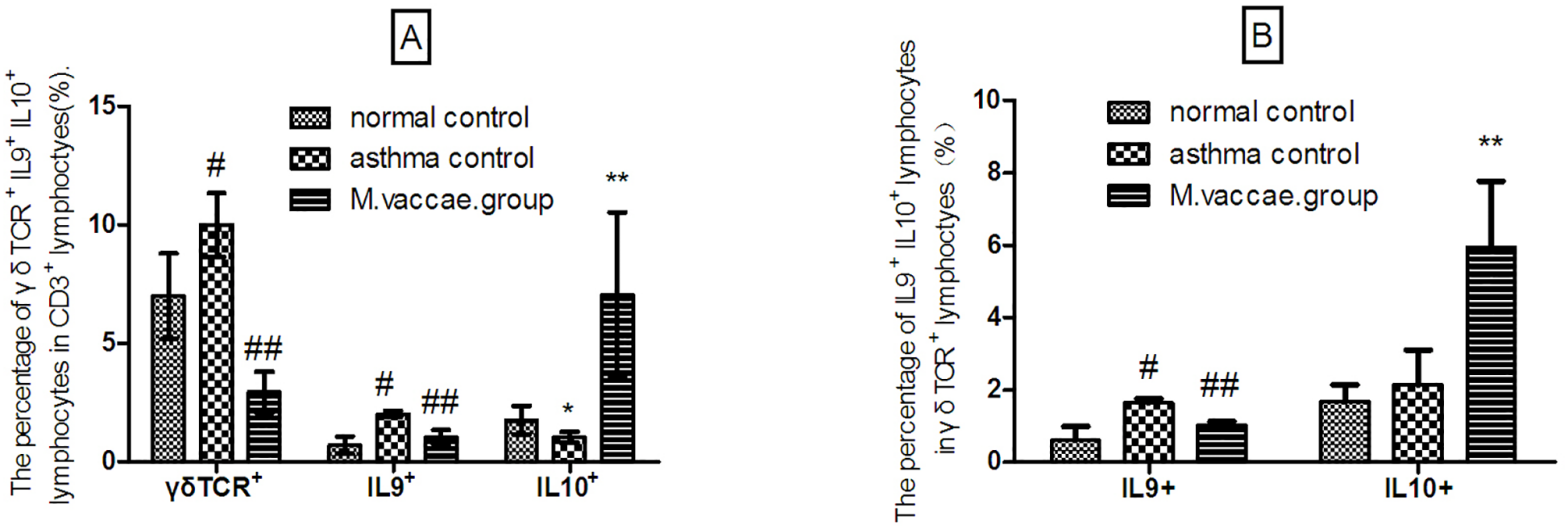
Flow cytometry results. #*P* < 0.001, compared with the normal control; ##*P* < 0.001, compared with the asthma control; *P < 0.05, compared with the normal control; **P < 0.01, compared with the asthma control.

**Fig 6:**
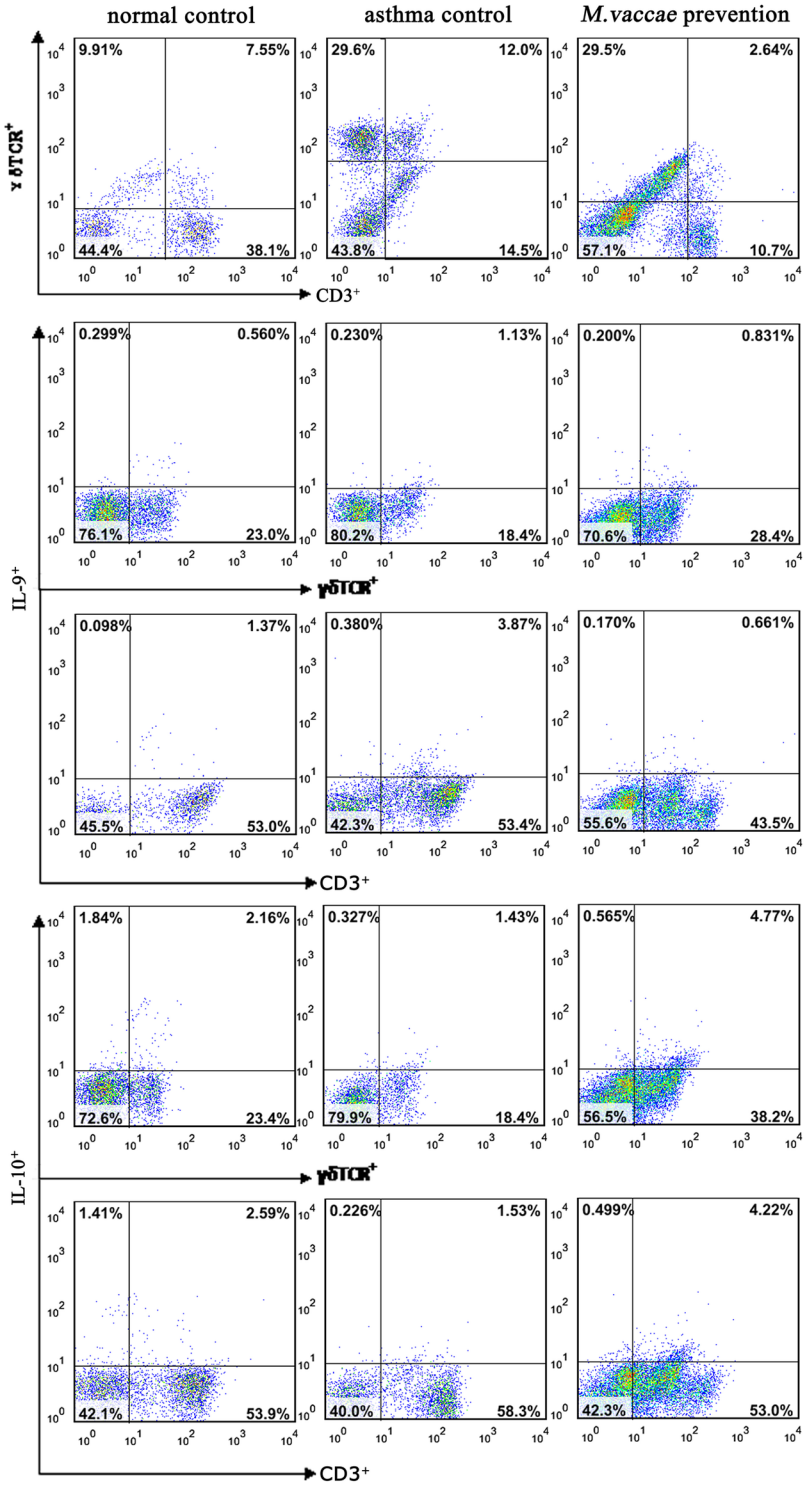
Flow cytometry images.

The percentage of γδTCR^+^CD3^+^, IL-9^+^CD3^+^, IL-9^+^γδT lymphocytes was higher, whereas the percentage of IL-10^+^ CD3^+^ lymphocytes was lower in the asthma control group than in the normal control group (*P* < 0.001). The percentages of IL-10^+^γδT lymphocytes were similar in the asthma and normal control groups. *M*. *vaccae* nebulization decreased the percentage of γδTCR^+^CD3^+^, IL-9^+^CD3^+^, IL-9^+^γδT lymphocytes (*P* < 0.001) and increased the percentage of IL-10^+^ CD3^+^, IL-10^+^γδT lymphocytes (*P* < 0.01) significantly (Figs 5A, 5B and 6).

## Discussion

In the present study, we found that *M*. *vaccae* nebulization could alleviate airway inflammation, reduce mucus secretion, and decrease airway hyperresponsiveness. It also sharply reduced the concentrations of IL-9 and OVA-specific IgE in the BALF. The present study also showed that IL-9^+^ lymphocytes in the lung tissue were diminished while IL-10^+^ lymphocytes increased substantially. This demonstrated that *M*. *vaccae* nebulization can protect against bronchial asthma by regulating Th9 cells. Moreover, the results illustrated that pulmonary γδT and IL-9^+^γδT cells increased considerably in asthmatic compared with normal mice, demonstrating that γδT cells may promote inflammation by IL-9 secretion. γδT cells may be the main source of IL-9, whereas there was no difference in the amount of secreted γδT cells suppressing cytokine IL-10 between asthmatic and normal mice, proving that the anti-inflammatory effect of γδT cells may not occur through IL-10 secretion. However, pulmonary γδT and IL-9^+^γδT cells decreased while IL-10^+^γδT cells increased considerably in mice nebulized with *M*. *vaccae*, demonstrating that *M*. *vaccae* nebulization can play an anti-inflammatory role by inhibiting the secretion of IL-9 from γδT, and inducing γδT cells to secrete IL-10; γδT cells dominantly expressed IL-10. Thus, γδT cells may be a member of the Th9 cell family.

The current study has overcome the limitations of routine intramuscular administration of *M*. *vaccae*, and mimicked BCG vaccination methods (immunity is developed 4–6 weeks after BCG vaccination in humans), as the asthmatic model was established 4 weeks after *M*. *vaccae* nebulization. It is known that natural killer (NK) T cells contribute to the non-specific (heterologous) beneficial effects of BCG vaccination [26]. Another study showed that NK T cells and Vδ2 γδ T cells are the key populations producing interferon-γ in response to BCG immunization [27]. This suggests that both NK T cells and γδ T cells participate in the mechanisms of BCG immunization. In addition, NK T cells are activated by BCG-stimulated dendritic cells mediated by IL-12 [28], and the IL-21 produced by activated NK T cells induces the programmed cell death of IgE-producing B-cells [29]. In the present study, we found that the concentrations of OVA-specific IgE in the BALF of asthmatic mice reduced sharply after nebulization of *M*. *vaccae*; however, the detailed mechanism contributing to this effect remains to be investigated.

In conclusion, we determined that *M*. *vaccae* nebulization could protect against bronchial asthma by alleviating airway inflammation and decreasing airway hyperresponsiveness. The nebulization method has several advantages, as it is simple and easy to apply with few side effects. Nebulization can be stopped immediately if discomfort is experienced, and asthma sufferers can operate it at home. However, many more experiments are required to provide theoretical support of this method prior to clinical application.
